# Biohythane Production in Hydrogen-Oriented Dark Fermentation of Aerobic Granular Sludge (AGS) Pretreated with Solidified Carbon Dioxide (SCO_2_)

**DOI:** 10.3390/ijms24054442

**Published:** 2023-02-23

**Authors:** Joanna Kazimierowicz, Marcin Dębowski, Marcin Zieliński

**Affiliations:** 1 Department of Water Supply and Sewage Systems, Faculty of Civil Engineering and Environmental Sciences, Bialystok University of Technology, 15-351 Bialystok, Poland; 2 Department of Environment Engineering, Faculty of Geoengineering, University of Warmia and Mazury in Olsztyn, 10-720 Olsztyn, Poland

**Keywords:** biohydrogen, biohythane, aerobic granular sludge (AGS), pre-treatment, solidified carbon dioxide (SCO_2_), anaerobic digestion, process optimization

## Abstract

Though deemed a prospective method, the bioconversion of organic waste to biohydrogen via dark fermentation (DF) has multiple drawbacks and limitations. Technological difficulties of hydrogen fermentation may, in part, be eliminated by making DF a viable method for biohythane production. Aerobic granular sludge (AGS) is a little-known organic waste spurring a growing interest in the municipal sector; its characteristics indicate the feasibility of its use as a substrate for biohydrogen production. The major goal of the present study was to determine the effect of AGS pretreatment with solidified carbon dioxide (SCO_2_) on the yield of H_2_ (biohythane) production during anaerobic digestion (AD). It was found that an increasing dose of SCO_2_ caused an increase in concentrations of COD, N-NH_4_^+^, and P-PO_4_^3−^ in the supernatant at the SCO_2_/AGS volume ratios from 0 to 0.3. The AGS pretreatment at SCO_2_/AGS ratios within the range of 0.1–0.3 was shown to enable the production of biogas with over 8% H_2_ (biohythane) content. The highest yield of biohythane production, reaching 481 ± 23 cm^3^/gVS, was obtained at the SCO_2_/AGS ratio of 0.3. This variant produced 79.0 ± 6% CH_4_ and 8.9 ± 2% H_2_. The higher SCO_2_ doses applied caused a significant decrease in the pH value of AGS, modifying the anaerobic bacterial community to the extent that diminished anaerobic digestion performance.

## 1. Introduction

Taking into account the increasing global demand for energy and the necessity of implementing the principles of sustainable development in practice, a justified need emerges to search for new, alternative, and clean energy carriers [1]. Their features, available production technologies, and environmental aspects of use mean that bioethanol, biomethane and above all biohydrogen (H_2_) may be deemed as the fuels of the future. H_2_ is considered one of the most cost-effective, versatile, and environmentally neutral fuels [2,3]. Its calorific value is high, reaching 122 MJ/kg, which is almost three times higher than that of oil [4]. It can be used in direct combustion processes, cogeneration systems, fuel cells for electricity production, or hydrogenation processes of conventional fuels [5]. The final outcome of its energy conversion is water vapor, which is of great importance considering the need to reduce greenhouse gas emissions into the atmosphere [6]. H_2_ may be produced in biological processes from biodegradable organic waste, which is in line with the assumptions of the closed-loop economy and energy recycling [7]. The potential of using one-stage hydrogen dark fermentation (DF) may be limited by incomplete biodegradation of biowaste. For this reason, many other individual and integrated processes are analyzed to improve technological and economic efficiency. These include photofermetation (PF) carried out by bacteria or microalgal direct biophotolysis (DB), as well as other integrative approaches, including DF–H_2_/PF–H_2_, DF–H_2_/DF–CH_4_ or DF–H_2_/value-added products. These technological solutions have been proposed for enhancing the bioprocess economy/circular economy for the complete valorization of organic waste [8].

Given the advantages of H_2_, on 8 July 2020, the European Commission published the “Hydrogen Strategy for a Climate-Neutral Europe” [9]. This strategy assumes that green H_2_ will be the foundation of the zero-emission economy and contribute to achieving the goals of the ambitious European Green Deal [10]. The widespread production and exploitation of H_2_ will serve as the means to decarbonize the economy and will also enable storing energy and balancing systems based on renewable energy [11]. The strategy for achieving EU climate neutrality also assumes an increase in its share in the EU energy mix from the current 2% to approx. 13–14% in the upcoming years [12].

Although H_2_ can be produced using various methods, the bioconversion of organic matter via dark fermentation (DF) has been considered to be the most economically and environmentally viable technology, deploying municipal, agricultural, and industrial waste as the initial substrate [13]. However, H_2_ production via DF has many weaknesses and limitations. First of all, it requires high energy inputs for inoculum preparation [14], high values of the organic load rate (OLR), and short hydraulic retention time (HRT) which implies operational problems related to foaming and diminishes the effectiveness of pollutant biodegradation [15]. Its other drawbacks include: the use of a multistage process line [16], low efficiency of H_2_ production [17], impurities and the presence of accompanying gases [18], a narrow range of required environmental parameters [19], and, in the long term, difficulties in maintaining an appropriate taxonomic composition of bacterial microflora [20]. Biohydrogen production can also be suppressed or even inhibited due to some specific characteristics of wastewater, sewage sludge, or organic waste, such as insufficient buffering capacity [21], nutrient imbalance [22], and the presence and development of microbial populations that can consume biohydrogen and/or produce CH_4_ [23].

The limitations presented above directly limit the applicability and possibility of commercialization of the H_2_ production technology via DF. A solution that can minimize or eliminate the technological difficulties of hydrogen fermentation may be orienting DF towards biohythane production [24]. Biohythane is an alternative biofuel containing from 8% to 30% H_2_ and accompanying gases, i.e., CH_4_ and CO_2_ [25,26]. The advantages and possibilities of the relatively simple production and versatile use of this energy carrier have spurred great interest in the fuel sector and automotive industry in the USA [27]. The studies carried out so far have shown the feasibility of producing biohydrogen and biohythane using a broad range of biodegradable organic waste from agriculture, the food industry, and the municipal sector [28,29]. H_2_ may be a promising alternative energy source in biotechnologically oriented methanotrophic projects, such as the production of high-quality feed protein, allowing biomass yield from CH_4_ to be maximized [30]. On the other hand, it has also been proven that biowaste sugars and CH_4_ and CO_2_ gases can be used to produce environmentally friendly biofuels such as H_2_ or methanol [31].

However, there are no studies on the targeted production of H_2_ during the anaerobic digestion (AD) of activated granular sludge (AGS). Wastewater treatment technologies based on the use of AGS compete in many respects with those based on the conventional activated sludge (CAS). AGS-based treatments are becoming increasingly popular, as evidenced by the dynamically growing number of installations operated on a commercial scale [32]. The compact and complex structure of AGS improves sedimentation capacity and allows for effective retention of biomass in bioreactors, which reduces both the number of digesters needed and investment costs [33]. The multispecies structure of granules often enables simultaneous and effective nitrification, denitrification, and bioremoval of phosphorus [34].

A prerequisite for an efficient AD is to increase the availability of organic substances contained in the substrate for the consortia of fermentative bacteria [35]. Due to the stable and complex structure of granules, this is of particular importance in the case of AGS fermentation [36]. Pretreatment methods are deployed in order to improve the efficiency of degradation of complex organic compounds and accelerate their transfer to the dissolved phase [37]. The use of solidified carbon dioxide (SCO_2_) is a promising technique, as the SCO_2_ can be recovered during the upgrading and enrichment of biogas or in natural gas purification processes; however, this technique is poorly understood [38]. The sources of SCO_2_ and its production methods are directly in line with the assumptions of the circular economy and material recycling [39]. They also support the idea of reducing carbon dioxide emissions through its sequestration and use in a closed cycle [40]. The disintegrating effect of SCO_2_ on AGS is caused by an increase in the volume of water freezing in the cytoplasm, mechanical damage to the cell wall and membrane, osmotic shock, and destruction of cell organelles. This series of processes results in the transfer of intracellular substances to the dissolved phase of the fermentation medium [41].

There are no reports in the world literature on the use of SCO_2_ for AGS pretreatment prior to AD which aimed to produce biogas rich in H_2_. Therefore, this study may be considered pioneering and original as it produced surprising results and contributed to expanding knowledge in the field of biohydrogen production. Given the characteristics of AGS, and the results obtained so far regarding SCO_2_ use to disintegrate CAS, the main objective of the present study was to determine the effect of aerobic granular sludge (AGS) pretreatment with solidified carbon dioxide (SCO_2_) on the efficiency of H_2_ (biohythane) production during anaerobic digestion (AD). The study assessed the impact of the tested pretreatment method on the characteristics of AGS, changes in the concentrations of organic compounds and nutrients in the dissolved phase, as well as the taxonomic structure of anaerobic bacterial community, and finally, production kinetics and qualitative composition of biogas. Empirical optimization models were also developed to enable estimation of biohydrogen and biohythane yields.

## 2. Results and Discussion

### 2.1. Stage 1

**Figure 1:**
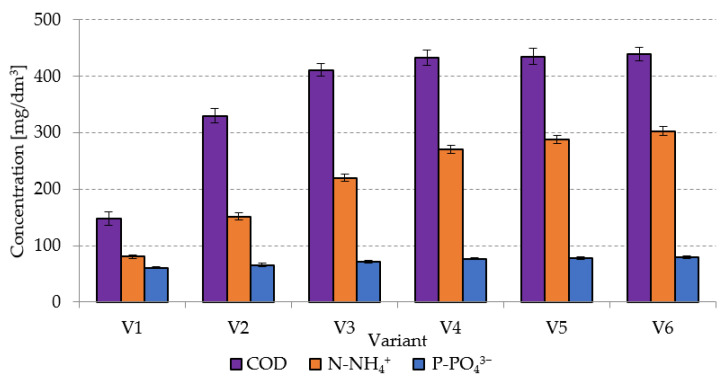
Changes in concentrations of organic compounds and nutrients in AGS supernatant caused by the pretreatment with SCO_2_.

In the case of the non-pretreated sludge (V1), the COD concentration in the supernatant was at 148 ± 12 mgO_2_/dm^3^ (Figure 1). In variants V2–V4, it was observed to increase from 330 ± 13 mgO_2_/dm^3^ in V2 to 433 ± 13 mgO_2_/dm^3^ in V4. Increasing the SCO_2_ dose in the subsequent variants did not cause any significant changes in COD concentrations in the supernatant (Figure 1), which remained at 435 ± 15 mgO_2_/dm^3^ in V5 and at 439 ± 12 mgO_2_/dm^3^ in V6.

Studies conducted thus far have not entailed AGS pretreatment with the use of SCO_2_. In turn, the viability of this pretreatment method has been confirmed in the case of CAS, by means of, i.a., FTIR analysis [40]. The increase in dissolved COD concentration has been proved to be related to the breaking of CAS structures followed by the degradation of single cells of microorganisms [42]. Damage to CAS cell structures upon exposure to SCO_2_ may cause an increase in concentrations of dissolved COD, ammonia nitrogen, proteins, orthophosphates, and molecular material in the supernatant [43]. This pretreatment additionally resulted in the improved CAS susceptibility to dehydration and in the increased supernatant turbidity [44]. Other researchers [45], who analyzed the impact of freezing/thawing on food waste, demonstrated a more than twofold increase in COD concentration in the supernatant. Zawieja (2018) [46] also noted a significant increase in COD concentration in the supernatant, ranging from 119 mgO_2_/dm^3^ to 296 mgO_2_/dm^3^ [46]. In a study conducted by Machnicka et al. (2019) [40], pretreatment with SCO_2_ at the SCO_2_/CAS volume ratio of 0.25 caused COD concentration to increase from 63 mgO_2_/dm^3^ in the raw sludge to 205 mgO_2_/dm^3^ in the pretreated sludge. In turn, at the SCO_2_/CAS ratio of 1.0, the COD concentration in the supernatant peaked to 889 mgO_2_/dm^3^ [40]. In another study [43], the highest COD concentrations in the supernatant, ranging from 490.6 ± 12.9 to 510.5 ± 28.5 mg/dm^3^, were determined at SCO_2_/CAS ratios from 0.3 to 0.5 applied in the pretreatment of dairy CAS. The cited authors emphasized that statically significant changes in COD concentration were no longer observed at the ratio exceeding 0.3. In the crude CAS supernatant, the COD concentration reached 400.5 ± 23.8 mg/dm^3^ [43].

Analyses made in the present study for the supernatant of crude AGS showed that it contained 80.45 ± 2.8 mg N-NH_4_^+^/dm^3^ and 61.1 ± 2.1 mg P-PO_4_^3−^/dm^3^. The increasing SCO_2_ dose caused a significant increase in the concentrations of both N-NH_4_^+^ and P-PO_4_^3−^ determined in variants V1–V4. In V2, the N-NH_4_^+^ concentration reached 152 ± 6.5 mg/dm^3^ and that of P-PO_4_^3−^ reached 65.8 ± 3.0 mg/dm^3^. (Figure 1). In V4, the respective values were at 271 ± 7.5 mg/dm^3^ and 76.9 ± 2.1 mg/dm^3^ (Figure 1). Increases noted in the concentrations of these compounds in V5 and V6 were not statistically significant. The increase in concentrations of ammonia nitrogen and orthophosphates in the supernatant was ascribed to degradation of organic nitrogen and phosphorus as a result of the hydrolytic activity of enzymes contained in protoplasts of microorganisms and released upon their cell structure damage by SCO_2_ [47]. In a study conducted by Montusiewicz et al. (2010) [48], the freezing/thawing pretreatment of CAS ensured an increase in the N-NH_4_^+^ concentration in the supernatant from 94.0 mg/dm^3^ in the raw sludge to 130.9 mg/dm^3^ in the pretreated CAS as well as over twofold increase in P-PO_4_^3−^ concentration from 86.4 mg/dm^3^ in the control sample to 185.2 mg/dm^3^ in the pretreated sludge In another research [43], the pretreatment of dairy CAS at the SCO_2_/CAS volume ratio of 0.5 caused a N-NH_4_^+^ concentration peak in the supernatant to 185.9 ± 11.1 mg/dm^3^, compared to the value of 155.2 ± 10.2 mg/dm^3^ determined in the raw supernatant. Similarly, an increasing SCO_2_ dose caused the P-PO_4_^3−^ concentration to increase from 198.5 ± 23.1 to 300.6 ± 35.9 mg/dm^3^ [43]. In the study conducted by Zawieja (2019) [49], the N-NH_4_^+^ concentration in the supernatant of crude CAS approximated 43 mg/dm^3^ and increased successively along with an increasing SCO_2_ dose to ca. 102 mg/dm^3^ at the SCO_2_/CAS volume ratio of 0.75/1.0 [49].

### 2.2. Stage 2

#### 2.2.1. Biogas, CH_4_, and H_2_ Production

**Figure 2:**
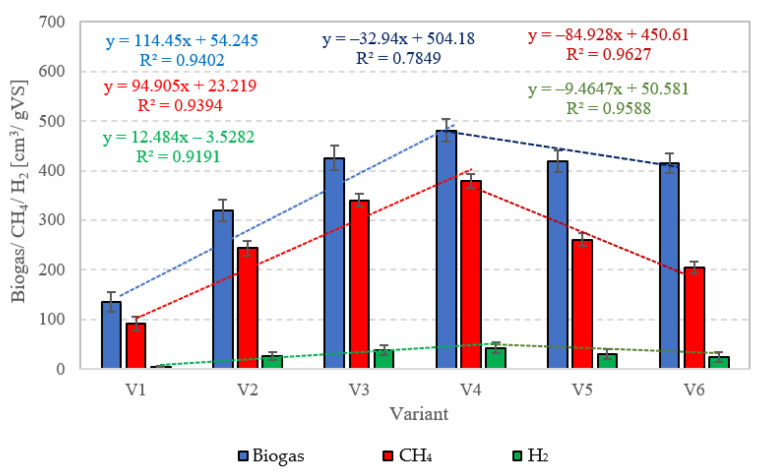
Production of biogas, CH_4_, and H_2_ in particular technological variants.

**Figure 3:**
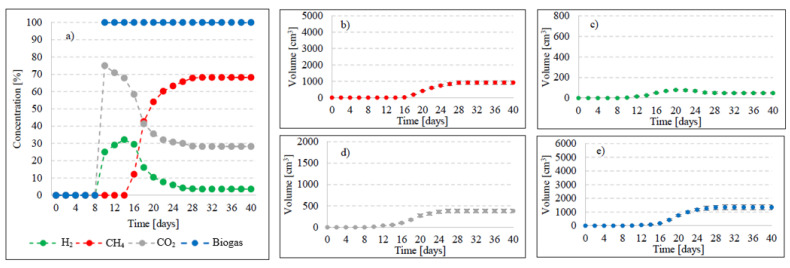
Changes in concentrations of biogas fractions in V1 (**a**); total volumes of CH_4_ (**b**), H_2_ (**c**), CO_2_ (**d**), and biogas (**e**).

In this experimental stage, variants V1–V4 produced significant increases in the yield of biogas and its main fractions. Biogas yield of crude AGS (V1) was 135 ± 20 cm^3^/gVS (Figure 2), whereas the reaction rate constant reached r = 60.72 cm^3^/d (Table 1). In V1, the initiation of the metabolic activity of anaerobes was observed after 10 days of retention in respirometers. At the initial stage of anaerobic digestion, CO_2_ and H_2_ were the main biogas fractions, with their concentrations reaching 75% and 25% on day 10 and 67.9% and 32.1% on day 14, respectively (Figure 3). On day 16, CH_4_ was detected in biogas. Its percentage content reached 11.5% and was observed to increase successively throughout the process. On day 20, H_2_ production ceased at 79 cm^3^, and its volume ultimately dropped to 49 cm^3^ at the end of experiment (Figure 3). The volume of CO_2_ stabilized at 381 cm^3^ on day 30, whereas that of CH_4_ stabilized at 920 cm^3^ on day 32. The ultimate biogas composition was as follows: 3.6% of H_2_, 28.2% of CO_2_, and 68.2% of CH_4_ (Figure 3, Table 2).

**Figure 4:**
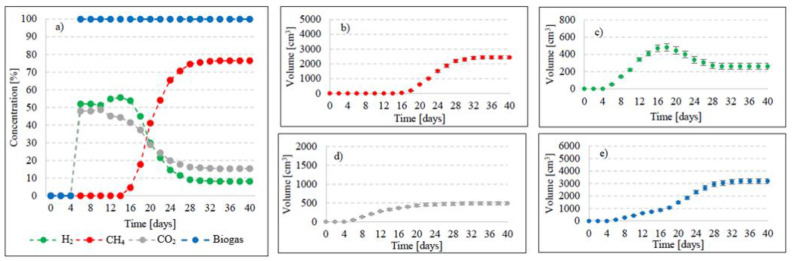
Changes in concentrations of biogas fractions in V2 (**a**); total volumes of CH_4_ (**b**), H_2_ (**c**), CO_2_ (**d**), and biogas (**e**).

**Figure 5:**
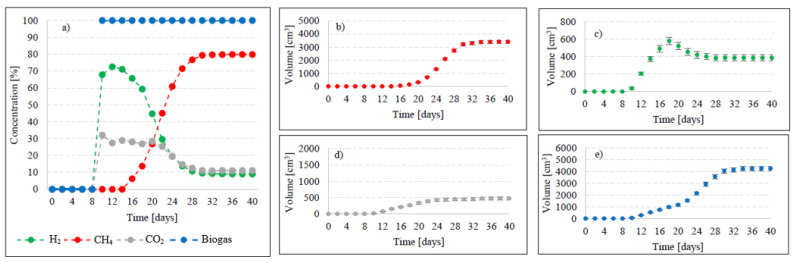
Changes in concentrations of biogas fractions in V3 (**a**); total volumes of CH_4_ (**b**), H_2_ (**c**), CO_2_ (**d**), and biogas (**e**).

**Figure 6:**
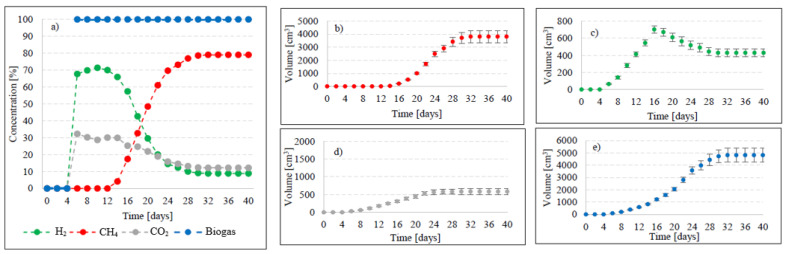
Changes in concentrations of biogas fractions in V4 (**a**); total volumes of CH_4_ (**b**), H_2_ (**c**), CO_2_ (**d**), and biogas (**e**).

Variants V2–V4 resulted in the production of biohythane, namely biogas with an admixture of gaseous H_2_ exceeding 8% [26]. The H_2_/(H_2_ + CH_4_) ratio reached 0.10 ± 0.01 in these variants. The highest yields of biohythane, CH_4_, and H_2_ were obtained in V4 and reached 481 ± 23 cm^3^/gVS, 380 ± 14 cm^3^/gVS, and 42.76 ± 11 m^3^/gVS, respectively (Figure 2, Table 2). The production rates were as follows: 723.61 cm^3^/d biohythane, 424.71 cm^3^/d CH_4_, and 30.71 cm^3^/d H_2_ (Table 1). In V2, the onset of production of biogas containing 52.0% of H_2_ and 48% of CO_2_ was noted on day 6 of anaerobic digestion (Figure 4). Similar ratios of biogas fractions were observed within the subsequent 10 days. On day 16, CH_4_ was detected in the biogas and its concentration reached 4.6%. Ever since, it became the major biogas fraction and its concentration stabilized at 76.5% after 34 days of the process (Figure 4). A significant increase in H_2_ volume to the value of 482 cm^3^ was observed until day 18 of anaerobic digestion. At the end of the process, the ultimate volume reached 262 cm^3^, which accounted for 8.2% of the total biogas composition (Figure 4). In V3, the H_2_ content in the biogas ranged from 65.7% to 72.6% between days 10 and 16 of anaerobic digestion (Figure 5). The CH_4_ concentration of 6.3% was determined on day 16 of the process. In the subsequent days of AD, this biogas fraction was produced most intensively, which had a direct impact on the qualitative composition of gaseous metabolites of anaerobes. At the end of digestion, the biogas contained 9% of H_2_, 79.9% of CH_4_, and 11.1% of CO_2_ (Figure 5, Table 2). Analogous course of AD was observed in V4. Between days 6 and 14, the content of H_2_ fell within a narrow range from 65.9% to 71.3%, whereas that of CO_2_ was from 29.9% to 32.3% (Figure 6). The highest H_2_ content in the biogas, peaking to 701 cm^3^, was determined on day 16 of AD (Figure 6). In the consecutive days of the process, the volume of H_2_ produced dropped to 428 cm^3^. In turn, a successive increase in CH_4_ production in the subsequent days directly affected biogas characteristics, which ultimately contained 8.9% of H_2_, 79.0% of CH_4_, and 12.1% of CO_2_ (Figure 6, Table 2).

**Figure 7:**
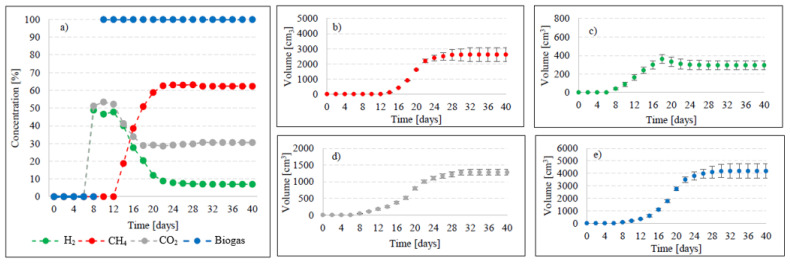
Changes in concentrations of biogas fractions in V5 (**a**); total volumes of CH_4_ (**b**), H_2_ (**c**), CO_2_ (**d**) and biogas (**e**).

**Figure 8:**
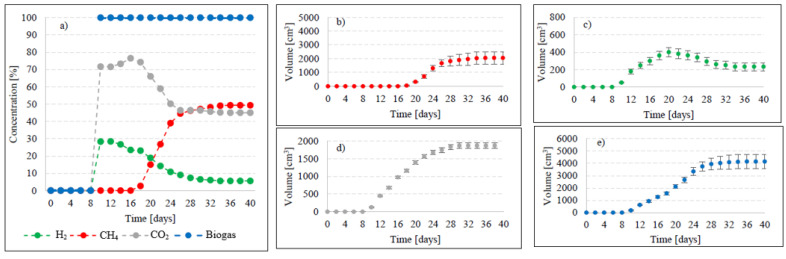
Changes in concentrations of biogas fractions in V6 (**a**); total volumes of CH_4_ (**b**), H_2_ (**c**), CO_2_ (**d**), and biogas (**e**).

Variants V5–V6 brought about AD yield reduction and a significant decrease in CH_4_ and H_2_ concentrations, whose values reached 418 ± 22 cm^3^/gVS biogas, 261 ± 10 cm^3^/gVS CH_4_, and 29.39 ± 10 cm^3^/gVS H_2_ in V5 (Figure 2), as well as 415 ± 20 cm^3^/gVS biogas, 205 ± 12 cm^3^/gVS CH_4_, and 23.32 ± 10 cm^3^/gVS H_2_ in V6 (Figure 2, Table 2). In V5, until day 12 of AD, the CO_2_ turned out to be the major biogas fraction, with its content in the biogas ranging from 51.2% to 53.4% (Figure 7). In the same time span, the content of H_2_ in the biogas ranged from 46.6% to 48.8%, which was significantly lower compared to that noted in V2–V4. The presence of CH_4_ was noted on day 14 of AD, when its volume reached 112 cm^3^ (18.7%), (Figure 7). At the end of digestion, the total CH_4_ volume was 2610 cm^3^, and its percentage content in the biogas reached 62.4%. The content of CO_2_ accounted for 30.6%, whereas that of H_2_ was 7% (Figure 7). In V6, biogas production was initiated on day 10 of AD (Figure 8). Since the very beginning, CO_2_ was the major biogas fraction, with its content in the biogas ranging from 66% to 76.5% until day 20 of AD (Figure 8). This variant produced the lowest H_2_ content in the biogas, which decreased from 28.5% on day 12 to 5.6% at the end of AD. The total volume of produced CH_4_ was 2050 cm^3^, and its content in the biogas ultimately accounted for 49.4% (Figure 8, Table 2).

Productivity of biohydrogen and biohythane from AGS pretreated with SCO_2_ has not been explored yet. Studies conducted so far have only determined the feasibility of producing typical biogas. Bernat et al. (2017) [50] achieved biogas yield ranging from 318.5 cm^3^/gVS at OLR = 6 gVS/cm^3^·d to 408.9 cm^3^/gVS at OLR = 2 gVS/cm^3^·d and concluded that the yield decreased along with OLR increase. In turn, the CH_4_ concentration in the biogas ranged from 56.7% to 59.5%. These values were significantly lower compared to those achieved in the present study. Another study demonstrated that biogas productivity from AGS was 1.8-fold lower than from CAS [50]. In turn, Cydzik-Kwiatkowska et al. (2022) [37] applied the ultrasound pretreatment of AGS. After 0.5, 4.0, and 8.0 min of disintegration, the biogas yield approximated 400 cm^3^/gVS, 420 cm^3^/gVS, and 455 cm^3^/gVS, respectively. Hence, a significant improvement was achieved in AD effectiveness compared to the nonsonicated AGS, which allowed ca. 375 cm^3^/gVS biogas to be produced [37]. A study conducted by Xiao and Liu (2009) [51] showed the impact of sewage sludge pretreatment on H_2_ yield. The alkaline pretreatment at the initial pH = 11.5 enabled H_2_ yield of 11.68 cm^3^/gVS and heat treatment ensured 8.62 cm^3^/gVS H_2_, whereas ultrasound pretreatment allowed the production of 3.83 cm^3^/gVS H_2_. No sludge pretreatment at pH = 7 resulted in H_2_ production at 1.21 cm^3^/gVS. In another study [52], municipal CAS was pretreated prior to AD using SCO_2_. In the most effective variant, biogas yield was 49% higher compared to that obtained with raw CAS [52]. Zawieja (2019) [49] also investigated the effect of pretreatment using SCO_2_ on the course of methane fermentation of CAS. At the SCO_2_ to CAS volume ratio of 0.55/1, they achieved biogas yield of 620 cm^3^/gVS and CH_4_ concentration in the biogas reaching ca. 78% [49].

#### 2.2.2. pH, FOS/TAC, and Bacterial Community Structure

In V1, the pH value reached 7.78 ± 0.1 and decreased successively as a result of the AGS pretreatment with SCO_2_, reaching 6.92 ± 0.1 in V4 (Table 3). A further increase in the SCO_2_ dose in V5 and V6 reduced the pH value of AGS to 6.41 ± 0.1 and 6.33 ± 0.1, respectively. During CO_2_ sublimation, its part was most likely dissolved in the supernatant. CO_2_ is well soluble in aqueous solutions, and its solubility at a temperature of 25 °C reaches 2900 mg/dm^3^ [53]. Hydrogen ions H^+^, carbonate ions CO_3_^2−^, and bicarbonate ions HCO_3_^−^ are formed then, causing pH drop [54] and negatively affecting AD yield [48]. This phenomenon may contribute to the inhibition of methanogenesis and—to a lesser extent—the activity of hydrogen-producing bacteria which are more resistant to low pH values [55]. Hence, the pH values measured in digesters were a consequence of initial conditions after pretreatment with SCO_2_. In V1, after AD, the pH value decreased from 7.51 ± 0.1 to 7.21 ± 0.1 (Table 3). In V2–V4, the pH ranged from 7.08 ± 0.1 to 7.38 ± 0.1 and decreased after AD to the values ranging from 6.64 ± 0.1 to 6.88 ± 0.1 (Table 3). In V5 and V6, in which the pretreatment caused environment acidification, the pH value dropped significantly after AD, from 6.84 ± 0.1 to 6.32 ± 0.1 in V5 and from 6.79 ± 0.1 to 6.25 ± 0.1 in V6 (Table 3). These pH changes were reflected in the structure of anaerobic microorganisms and, consequently, in biogas production yield and composition. The pH value in the anaerobic digester is a key driver of process stability [56]. The present study results confirm findings from earlier research [49], which also demonstrated a decrease in pH values after pretreatment with SCO_2_ and a subsequent decline in AD effectiveness.

The values of the FOS/TAC ratio fell within a narrow range from 0.36 ± 0.03 in V1 to 0.43 ± 0.03 in V5 and V6 (Table 3). These differences were not statistically significant. The FOS/TAC ratio, used to assess AD stability, represents the ratio of volatile organic acids to the alkaline buffer capacity [57]. According to existing literature [58], the FOS/TAC values of a stable AD process range from 0.3 to 0.4. Hence, this condition was met in the present study. The FOS/TAC values exceeding 0.5 are indicative of operating conditions that are inappropriate for anaerobic microorganisms and cause biogas yield decline [57].

The bacteria (EUB338) turned out to be the prevailing consortium of microorganisms in all technological variants, with their percentage in the population of anaerobes ranging from 68 ± 11% in V6 to 70 ± 12% in V2 and V3 (Table 4). In V1–V4, the taxonomic structure of the population of fermentative bacteria was similar. The methanogenic archaea (ARC915) accounted for 23 ± 3 to 24 ± 5%, *Methanosarcinaceae* (MSMX860) for 11 ± 3% to 13 ± 4%, whereas *Methanosaeta* (MX825) for 6 ± 2 to 8 ± 4% of the total anaerobic bacteria community (Table 4). In V5 and V6, the contribution of archaea (ARC915) diminished to ca. 21%, whereas *Methanosarcinaceae* (MSMX860) accounted for 12 ± 3% in V5 and for 11 ± 5% in V6, and *Methanosaeta* (MX825) for 7 ± 3% in V5 and 6 ± 2% in V6 (Table 4). This structure of the anaerobic bacteria community could be due to environment acidification prior to fermentation caused by sludge pretreatment with SCO_2_, which has a significant impact on the environmental conditions and on the course and effectiveness of AD, since methanogenic bacteria are functionally active at the pH range from 6.5 to 7.5 [59].

In all experimental variants, the most abundant taxonomic group was *Methanosarcinaceae*, being facultative acetoclastic methanogens that may also use H_2_ and CO_2_ compounds to produce CH_4_ [60]. *Methanosaeta* are obligate acetoclastic methanogens known to use only acetate or electrons gained via direct interspecies electron transfer [60]. It is also speculated that hydrogenotrophic methanogens, such as *Methanobacterium* sp., were present in digesters during AD, which converted H_2_ and CO_2_ formed upon anaerobic digestion and CO_2_ dissolved in the substrate as a result of pretreatment with SCO_2_ into CH_4_ [61].

The often promoted biohythane production technologies are two-stage solutions, where H_2_ and CH_4_ are produced in separate anaerobic chambers [62]. This is justified by the required different optimal environmental conditions for communities of hydrogen bacteria and methanogenic archaea. However, the practical implementation of such technological systems is very limited due to the single-stage anaerobic process prevailing on a commercial scale. Two-stage digesters account for only 7% of all installations operated in Europe, which is determined primarily by economic and operational aspects [62]. For this reason, a justified direction of research is the development of a single-stage biohythane production technology. The work published so far shows that this is an entirely viable alternative [62]. The most important factor is to maintain balance in the structure of anaerobic bacteria, because in a single-stage biohythane production system, microorganisms of different groups compete at H_2_ production stage [62]. During DF, H_2_ produced by hydrogen bacteria is mainly consumed by methanogenic bacteria in a metabolic pathway called hydrogenotrophic methanogenesis; the reduction of carbon dioxide (CO_2_) produces CH_4_ according to the reaction (4H_2_ + CO_2_ → CH_4_ + 2H_2_O) [62]. The low content of H_2_ in biogas prevents the production of biohythane, because commercial hythane contains more than 8% H_2_ by volume [62]. Thus, the increase in H_2_ concentration in biogas depends on the rate of hydrogenotrophic methanogenesis [63]. On the other hand, inhibition of hydrogenotrophic methanogenesis can lead to a decrease in CH_4_ production, as this mechanism maintains a low H_2_ partial pressure state which is necessary for the metabolic reactions of methanogenic bacteria [63]. Therefore, to achieve the desired biohythane production in a single-stage system, it is crucial to overcome the conflict of H_2_ and CH_4_ production that is associated with hydrogenotrophic methanogenesis. Previous studies have shown that the taxonomic structure of microorganisms plays a key role in the bioprocess of biohythane production. It is related to the efficiency of degradation of organic substances and metabolic mechanisms leading to the production of final gaseous products. In previous works, it was proved that the production of biohythane was dominated by hydrogen bacteria of the species *Clostridium butyricum*, and that *Methanovbacterium beijiingense* and *Methanothrix soehngeni* were dominant for methanogenic bacteria [63]. In other works, the dominant hydrogen bacteria were *Clostridium pasteurianumi*, while the methanogenic genus *Methanosaeta* and *Methanobacterium* [63,64].

#### 2.2.3. Correlations and Empirical Models

**Figure 9:**
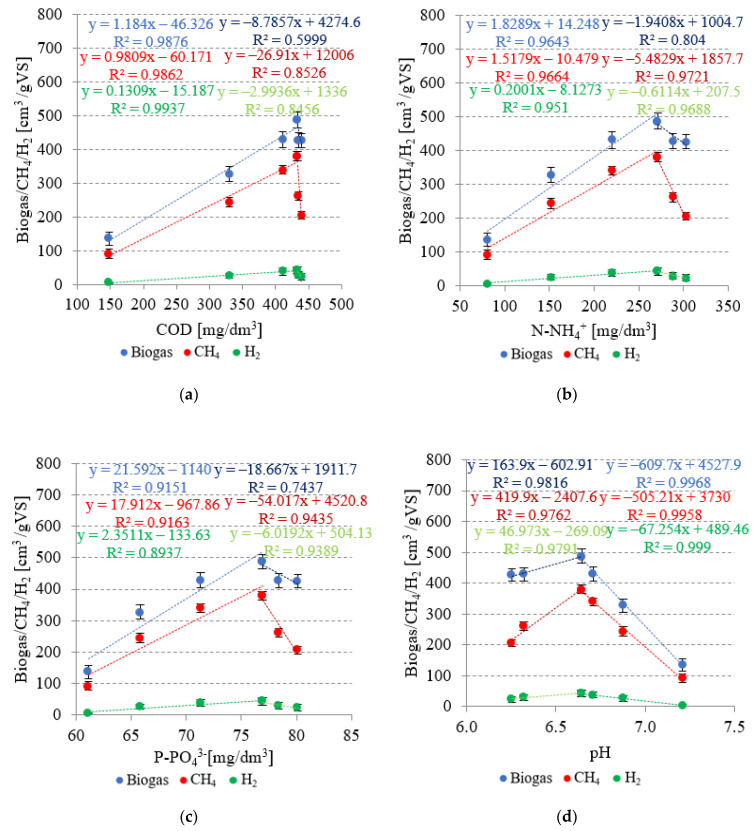
Correlations between: (**a**) COD, (**b**) N-NH_4_^+^, and (**c**) P-PO_4_^3−^ concentrations in the dissolved phase, (**d**) pH, and the yields of biogas, CH_4_, and H_2_.

In variants from V1 to V4, very strong positive correlations were found between the concentrations of COD, N-NH_4_^+^, and P-PO_4_^3−^ in the dissolved phase and biogas yield (Figure 9). The respective coefficients of determination reached R^2^ = 0.9876 (Figure 9a), R^2^ = 0.9643 (Figure 9b), and R^2^ = 0.9151 (Figure 9c). In the subsequent technological variants, increased SCO_2_ doses applied resulted in negative correlations between the concentrations of indicators monitored in the dissolved phase and biogas yield, i.e., R^2^ = 0.5999 for COD, R^2^ = 0.804 for N-NH_4_^+^, and R^2^ = 0.7437 for P-PO_4_^3−^ (Figure 9). Analogous phenomena were observed for the concentrations of COD, N-NH_4_^+^, and P-PO_4_^3−^ in the dissolved phase and the CH_4_ and H_2_ yields (Figure 9). In variants V1–V4, very strong positive correlations were found between COD (R^2^ = 0.9862), N-NH_4_^+^ (R^2^ = 0.9664), and P-PO_4_^3−^ (R^2^ = 0.9163) concentrations in the dissolved phase and CH_4_ yield, as well as among COD (R^2^ = 0.9937), N-NH_4_^+^ (R^2^ = 0.951) and P-PO_4_^3−^ (R^2^ = 0.8937) concentrations in the dissolved phase and H_2_ yield (Figure 9). The subsequent variants produced strong negative correlations, whose coefficients of determination reached R^2^ = 0.8526, R^2^ = 0.9721, and R^2^ = 0.9435 in the case of CH_4_ as well as R^2^ = 0.8456, R^2^ = 0.9688, and R^2^ = 0.9389 in the case of H_2_, respectively (Figure 9).

The V1–V4 variants were characterized by very strong negative correlations between pH after AD and production yields of biogas (R^2^ = 0.9968), CH_4_ (R^2^ = 0.9958), and H_2_ (R^2^ = 0.999) (Figure 9d). In these variants, the taxonomic structure of the population of fermentative bacteria was similar, whereas an increase in the yields of biogas, CH_4_, and H_2_ was due to the increased availability and improved biodegradability of the substrate after pretreatment. In variants V5 and V6, the availability and biodegradability were similar to those noted in V4, as indicated by concentrations of the analyzed indicators in the dissolved phase, whereas high SCO_2_ doses applied decreased the pH value, thereby inhibiting the populations of methanogenic bacteria. The methanogenic bacteria require neutral pH for their optimal metabolic activity and are highly sensitive to environmental changes [65].

**Figure 10:**
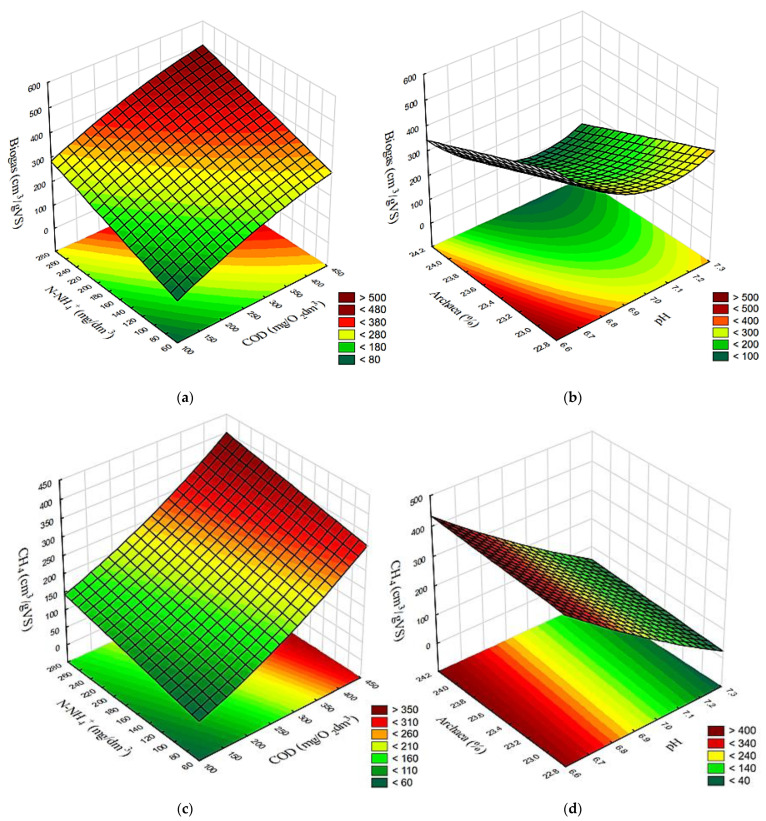

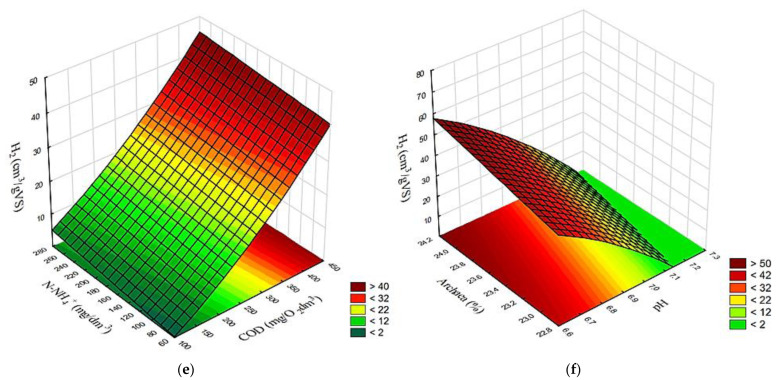
Surface correlation between concentrations of COD and N-NH_4,_ as well as pH and Archaea percentage, and yields of (**a**,**b**) biogas; (**c**,**d**) CH_4_; as well as (**e**,**f**) H_2_, respectively.

The results achieved in variants V1–V4 demonstrated a correlated surface effect of COD and N-NH_4_^+^ concentrations in the supernatant as well as the effect of pH and Archaea contribution in the populations of methanogens on the yields of biogas (Figure 10a,b), CH_4_ (Figure 10c,d), and H_2_ (Figure 10e,f).

The multiple regression method was deployed to develop empirical equations for estimating production yields of biogas, CH_4_, and H_2_. Only variants V1–V4 were considered in the estimation due to linear correlation. It was found that biogas and methane yields were statistically significantly affected by such predictors as COD concentration in the dissolved phase and the SCO_2_/AGS volume ratio. The postulated model of biogas yield (1) is characterized by an estimation error of ±1.5157 and reflects ca. 98.99% of changes in the process of biogas production (R^2^ = 0.9899). The CH_4_ yield model (2) reflects ca. 98.74% changes in the process of its production, with a coefficient of determination at R^2^ = 0.9874 and an estimation error of ±6.5299. In turn, the empirical model of H_2_ production (3) has a coefficient of determination at R^2^ = 0.9979 and an estimation error of ±0.7867.

(1) $$\mathrm{BIOGAS}=0.8397\mathrm{COD}+370.0324{\mathrm{SCO}}_{2}/\mathrm{AGS}+11.9713$$

(2) $${\mathrm{CH}}_{4}=0.6893\mathrm{COD}+313.4316{\mathrm{SCO}}_{2}/\mathrm{AGS}-10.7915$$

(3) $$H_{2}=0.1053\mathrm{COD}+27.4758{\mathrm{SCO}}_{2}/\mathrm{AGS}-10.8578$$

BIOGAS—biogas yield, cm^3^/gVS, 

CH_4_—methane yield, cm^3^/gVS, 

H_2_—hydrogen yield, cm^3^/gVS, 

COD—COD concentration in the supernatant, mgO_2_/dm^3^, 

N-NH_4_^+^—N-NH_4_^+^ concentration in the supernatant, mg/dm^3^, 

SCO_2_/AGS—volume ratio of SCO_2_ to AGS. 

The analysis of concentrations of selected indicators in the dissolved phase of the organic substrate subjected to pretreatment is one of the means used to evaluate the effectiveness of pretreatment processes [66]. Usually, concentrations of organic compounds are monitored to enable this assessment [67]. In some cases, it is feasible to develop reliable correlations and empirical models to estimate AD efficiency based on the presence of organic compounds in the dissolved phase [68]. Conducting analyses of this type is useful from a practical standpoint, as it eliminates the need to conduct more advanced measurements to estimate the effectiveness of the pretreatment methods applied.

## 3. Materials and Methods

### 3.1. Study Design

Experimental works were divided into two stages. Stage 1 (S1) involved AGS pretreatment using SCO_2_, whereas stage 2 (S2) included its anaerobic digestion (AD) focused on biohydrogen production. S1 and S2 were divided into 6 variants (V), differing in the volume ratio of SCO_2_ to AGS: V1—control; V2—1:10 (0.1); V3—1:5 (0.2); V4—1:3 (0.3); V5—1:2.5 (0.4); and V6—1:2 (0.5). The SCO_2_ doses applied were established based on the analysis of literature data [46,52] and the results of our previous research [43].

### 3.2. Materials

#### 3.2.1. AGS and Inoculum of the Anaerobic Sludge (AS)

The AGS was cultured under laboratory conditions and used conventional activated sludge (CAS) derived from the municipal wastewater treatment plant in Rajgród, Poland (53.99434 N, 22.76847 E) as the inoculum. The value of the population equivalent (PE) for this plant is 2500, its average capacity is 400 m^3^/d, and it operates based on Terrace-Flow technology with enhanced nutrient removal. The AGS was cultured in a sequencing batch reactor (SBR) [69], equipped in an oxygen diffuser and sensor, stirrer, and a discharge valve that enabled a decantation ratio of 0.33. The work cycle of the reactor spanned for 8 h, including 60 min of the filling and stirring phase, 270 min of the aeration phase, 15 min of the sedimentation phase, and 135 min of the decantation phase. The rotational speed of the agitator was 70 rpm. During the aeration phase, the concentration of dissolved oxygen in the reactor was kept at 2.5 mgO_2_/dm^3^. In the remaining phases of SBR work, dissolved oxygen concentration was kept at 0.3 mgO_2_/dm^3^. The SBR was fed with model wastewater that had the following composition: enriched broth (0.304 g/dm^3^), casein peptone (0.452 g/dm^3^), NH_4_Cl (0.242 g/dm^3^), CH_3_COONa (0.300 g/dm^3^), KH_2_PO_4_ (0.032 g/dm^3^), NaCl (0.014 g/dm^3^), K_2_HPO_4_ (0.080 g/dm^3^), MgSO_4_·7H_2_O (0.004 g/dm^3^), and CaCl_2_·6H_2_O (0.015 g/dm^3^). After 120 days of SBR exploitation, mature AGS was obtained that was used in the next experimental work.

The anaerobic sludge (AS) that was used as the inoculum in anaerobic respirometers originated from a closed fermentation chamber (CFC) with a volume of 7300 m^3^ and was exploited at the wastewater treatment plant in Białystok, Poland (53.16903 N, 23.08705 E). The CFC operates at a temperature of 35 °C, HRT = 21 days, and OLR = 2.0 kgVS/m^3^·d. Before being inoculated into experimental anaerobic reactors, the AS was pretreated with the heat shock method by heating to a temperature of 100 °C, then being retained at this temperature for 60 min to eradicate methanogenic archaea from the bacterial community and leave only the microorganisms of the acid (hydrogenic) phase of AD [70]. Table 5 presents the characteristics of CAS, AGS, and AS.

#### 3.2.2. SCO_2_

The pretreatment was conducted using SCO_2_ in the form of granules 3.0 ± 1.0 mm in diameter (Sopel Ltd., Bialystok, Poland). SCO_2_ is a natural, tasteless, odorless, nonflammable, and nontoxic product approved for contact with foods. Under atmospheric pressure, its sublimation occurs at −78.5 °C, whereas sublimation enthalpy is 573 kJ/kg, which makes it ca. 3.3 times more efficient than ice of the same volume [71].

### 3.3. Experimental Stations

**Figure 11:**
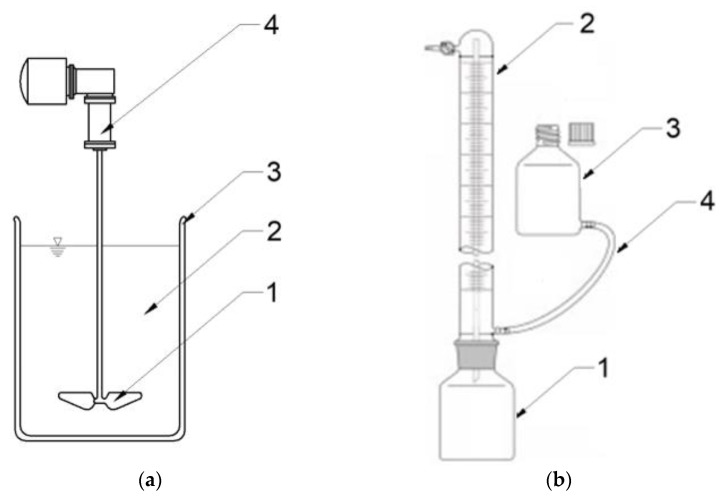
Schemes of research stations used in: (**a**) S1 (1—mechanical agitator, 2—AGS mixed with SCO2, 3—glass reactor, 4—drive); (**b**) S2—scheme of an eudiometer (1—reactor, 2—burette with an internal glass tube for gas transport, 3—pressure equalization tank, 3—connecting tube).

S1 was performed with a multistation mixing set (JLT 6, VELP Scientifica, Milano, Italy). AGS that was at a temperature of 20 °C was poured into reactors in single doses of 200 cm^3^, and then an assumed dose of SCO_2_ was added. The mixture was stirred with the yield of 50 rpm for 20 min. When SCO_2_ had been completely sublimated and its temperature had reached 20 °C, it was fed to AD reactors. Figure 11a presents the scheme of a research station used in S1. S2 was conducted in eudiometers (Hornik Ltd., Poznań, Poland) consisting of a 1000 cm^3^ reactor coupled through a joint with a 600 cm^3^ burette. Inside the burette there is a thin capillary through which gas escapes from the reactor and flows to the top of the burette. From here, it can be collected for analyses through a port with a valve. The gas emitted displaces the liquid from the burette, which flows through the hose to the connected equalization tank to equalize the pressure. Figure 11b presents the scheme of a research station used in S2. The reactors were inoculated with 200 cm^3^ of AS, and then AGS was fed in a dose ensuring the initial organic load rate of OLR = 10.0 gVS/dm^3^ [43]. Afterwards, they were purged with nitrogen for 3 min to remove oxygen, and then placed in a water bath at 37 °C. The volume of biogas produced was read out every day. The process was completed when no biogas volume increase was observed for five consecutive days.

**Figure 12:**
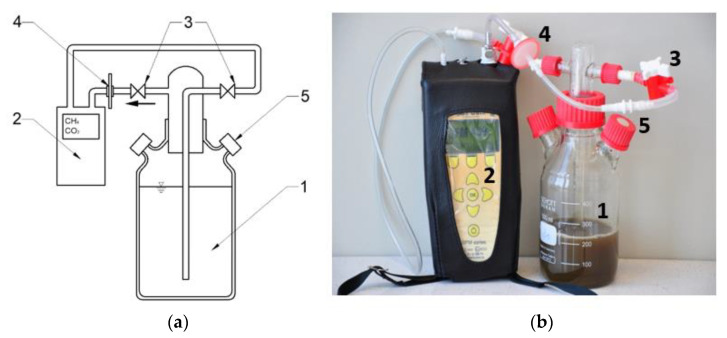
Diagram (**a**) and photograph (**b**) of a model respirometric fermentation chamber with a system for biogas quality analysis (1—fermentation chamber, 2—biogas composition analyzer, 3—valves cutting off biogas flow, 4—biogas dryer, 5—gas-tight port for sample collection for chromatographic analyses).

Next to eudiometers and to allow biogas volumes to be monitored, respirometers under the same conditions were used to enable the everyday control of the qualitative composition of biogas produced (Figure 12). Bioreactors were equipped with a closed-loop system where the analyzed biogas was returned to the fermentation chamber, enabling measurements of biogas composition. The measuring system consisted of a fermentation chamber, stub pipes, valves, a biogas dryer, and an analyzer. In addition, respirometers were equipped with ports that had rubber plugs, enabling gas-tight sample collection for chromatographic measurements.

### 3.4. Analytical Methods

Contents of TS, MS, and VS were determined with the gravimetric method. The TS content in the sludge was determined by drying to a constant weight at a temperature of 105 °C, followed by incineration at 550 °C. The loss after incineration constituted the VS value, accordingly to the Polish Standard PN-EN 15935:23022-01 [72]. Potentiometric method was used to determine pH. The total carbon (TC) content was determined based on a high-temperature degradation with infrared detection using a TOC multi-NC 3100 analyzer (Analytik Jena, Jena, Germany). Contents of total nitrogen (TN), ammonia nitrogen, orthophosphates, and COD in the supernatant were determined with the spectrophotometric method after mineralization using a Hach DR6000 spectrometer (Hach, Loveland, Dillon, CO, USA). The supernatant was obtained by AGS centrifugation using an MPW-251 laboratory centrifuge at 5000 rpm for 10 min (MPW Med. Instruments, Warsaw, Poland). The total protein content was estimated by multiplying the TN value by the conversion factor for proteins, or 6.25. The content of reduced sugars was determined colorimetrically at a wavelength of 600 nm using a DR 2800 spectrophotometer (Hach Lange, Düsseldorf, Germany), and the fat content was measured using the Soxhlet method in an extractor (Büchi, Flawil, Switzerland). The qualitative composition of biogas was assessed using a DP-28BIO analyzer (Nanosens, Wysogotowo, Poland), a gas data GMF430 analyzer (Gas Data, Coventry, UK), and a GC Agillent 7890A gas chromatograph (Agilent, Santa Clara, CA, USA) equipped in a thermal conductivity detector (TCD). The GC was equipped in two Hayesepa Q columns (80/100 mesh), two columns with molecular sieves (60/80 mesh), and a Porapak Q column (80/100) operating at a temperature of 70 °C. The temperature of the injection ports and detector was 150 °C and 250 °C, respectively. Helium and argon were used as carrier gases at a flow rate of 15 mL/min. Biogas quality was analyzed once per two days and at the end of the process, and measurements were conducted in eudiometers.

### 3.5. Molecular Methods

The molecular analysis aimed at determining the percentage content of anaerobic microorganisms in sewage sludge after AD by using the fluorescent in situ hybridization (FISH) technique. Four molecular probes were used for hybridization: a universal probe for Bacteria EUB338, a universal probe for Archaea ARC915, a probe oriented for Methanosarcinaceae MSMX860, and a probe oriented for Methanosaeta MX825. The samples were analyzed under an epifluorescent microscope (Nikon, Tokyo, Japan) with a 100× lens and total magnification at 1000×. The population numbers of the studied microorganisms were counted from cells stained with DAPI using Image Processing and Analysis in Java (ImageJ – https://imagej.net/software/imagej/) (LOCI, University of Wisconsin, Madison, WI, USA) [73].

### 3.6. Computation Methods

Biogas production rate (r) and reaction rate constants (k) were determined based on test data achieved with the iterative nonlinear regression method. The contingency coefficient φ^2^ < 0.2 was adopted as a measure of curve fit to the test data (Statistica 13.3 PL package (Statsoft, Inc., Tulsa, OK, USA)).

### 3.7. Statistical and Optimization Methods

Experiments were performed in three replicates. The statistical analysis of experimental results was carried using a STATISTICA 13.3 PL package (Statsoft, Inc., Tulsa, OK, USA). One-way analysis of variance (ANOVA) was conducted to determine differences between variances. The homogeneity of variance in groups was checked with the Levene test, whereas the significance of differences between the analyzed variables was established using the HSD Tukey test. In the tests, results were considered significant at α = 0.05. A stepwise multiple regression was applied to develop empirical equations, using the Statistica 13.3 PL package (Statsoft, Inc., Tulsa, OK, USA). Predictors of changes in the values of the estimated parameters were identified in mathematical models. Determination coefficients were used to verify the fit of the proposed model to empirical data.

## 4. Conclusions

In the case of SCO_2_/AGS volume ratios ranging from 0.1 to 0.3, the increasing SCO_2_ dose was observed to cause a proportional increase in COD, N-NH_4_^+^, and P-PO_4_^3−^ concentrations in the supernatant. The higher SCO_2_ dose applied had no significant effect upon increasing concentrations of the analyzed indicators in the supernatant.

This study demonstrated that AGS pretreatment using SCO_2_ at the SCO_2_/AGS ratios of 0.1–0.3 allows production of biohythane, i.e., biogas with over 8% hydrogen content. The highest yield of biohythane production, reaching 481 ± 23 cm^3^/gVS, was obtained after AGS with pretreatment at the SCO_2_/AGS ratio of 0.3. This variant produced 79.0 ± 6% CH_4_ and 8.9 ± 2% H_2_. Increasing the SCO_2_ dose caused no significant changes in the volumes of biogas and CH_4_. The biogas produced failed to meet the desired H_2_ content; hence there was no biohythane production. Optimization procedures demonstrated COD concentration as well as the SCO_2_/AGS ratio to be the significant predictors of changes in the values of the estimated parameters, i.e., biogas, CH_4_, and H_2_ yields.

It was also found that AGS’s pH decrease and environment acidification in the variants with SCO_2_ doses exceeding 0.3 decreased the percentage of methanogenic bacteria in the anaerobic bacterial community, which in turn significantly affected the ultimate biogas composition.

## Figures and Tables

**Table 1:** Rates (r) of production processes of biogas, CH_4_, and H_2_, and reaction rate constants (k).

Variant	Biogas		CH_4_		H_2_	
	r	k	r	k	r	k
	[cm^3^/d]	[1/d]	[cm^3^/d]	[1/d]	[cm^3^/d]	[1/d]
1	60.72	0.45	28.23	0.31	0.31	0.04
2	310.0	0.98	175.11	0.72	12.91	0.27
3	524.75	1.24	319.23	0.94	18.50	0.32
4	723.61	1.50	424.71	1.12	30.71	0.44
5	547.03	1.31	212.88	0.82	7.24	0.20
6	502.45	1.22	116.74	0.57	7.24	0.20

**Table 2:** Yields of biogas and its major fractions in particular technological variants.

		V1	V2	V3	V4	V5	V6
Biogas	cm^3^/gVS	135 ± 20	319 ± 22 *	426 ± 24 *	481 ± 23 *	418 ± 22	415 ± 20
CH_4_	%	68.2 ± 3	76.4 ± 4	79.9 ± 8	79.0 ± 6	62.4 ± 5	49.4 ± 6
	cm^3^/gVS	92.3 ± 14	244 ± 15	340 ± 13	380 ± 14	261 ± 13	205 ± 12
H_2_	%	3.6 ± 1	8.2 ± 2	9 ± 2	8.9 ± 2	7 ± 1	5.6 ± 1
	cm^3^/gVS	4.86 ± 2	26.16 ± 8	38.49 ± 10	42.76 ± 11	29.39 ± 10	23.32 ± 10
CO_2_	%	28.2 ± 4	15.4 ± 3	11.1 ± 2	12.1 ± 2	30.6 ± 5	45 ± 6
	cm^3^/gVS	38.08 ± 4	49.05 ± 11	47.3 ± 12	58.44 ± 10	128 ± 28	187 ± 34
H_2_/(H_2_ + CH_4_)		0.05 ± 0.01	0.10 ± 0.01	0.10 ± 0.01	0.10 ± 0.01	0.10 ± 0.01	0.10 ± 0.01

* biohythane.

**Table 3:** Changes in pH values upon pretreatment and AD, and FOS/TAC after AD.

	V1	V2	V3	V4	V5	V6
pH of AGS after pretreatment with SCO_2_	7.78 ± 0.1	7.49 ± 0.1	7.19 ± 0.1	6.92 ± 0.1	6.41 ± 0.1	6.33 ± 0.1
pH of AGS + inoculum	7.51 ± 0.1	7.38 ± 0.1	7.23 ± 0.1	7.08 ± 0.1	6.84 ± 0.1	6.79 ± 0.1
pH after AD	7.21 ± 0.1	6.88 ± 0.1	6.71 ± 0.1	6.64 ± 0.1	6.32 ± 0.1	6.25 ± 0.1
FOS/TAC	0.36 ± 0.03	0.37 ± 0.02	0.38 ± 0.03	0.40 ± 0.04	0.43 ± 0.03	0.43 ± 0.03

**Table 4:** Microbial taxonomy in particular experimental variants.

Taxonomic Group	V1	V2	V3	V4	V5	V6
Bacteria (EUB338)	69 ± 10	70 ± 12	70 ± 12	69 ± 12	69 ± 10	68 ± 11
Archaea (ARC915)	24 ± 5	23 ± 3	23 ± 6	23 ± 10	21 ± 8	21 ± 7
*Methanosarcinaceae* (MSMX860)	11 ± 3	12 ± 4	13 ± 3	13 ± 4	12 ± 3	11 ± 5
*Methanosaeta* (MX825)	6 ± 2	7 ± 2	7 ± 3	8 ± 4	7 ± 3	6 ± 2

**Table 5:** Characteristics of CAS, AGS, and AS used in the experiments.

Indicator	Unit	CAS	AGS	AS
pH	-	7.64 ± 0.1	7.78 ± 0.1	7.26 ± 0.2
Dry matter (TS)	[%]	4.3 ± 0.1	7.44 ± 0.1	3.38 ± 0.1
Organic dry matter (VS)	[%TS]	81.37 ± 2.4	90.2 ± 1.1	66.4 ± 2.9
Mineral dry matter (MS)	[%TS]	18.63 ± 1.1	9.8 ± 1.2	33.6 ± 1.5
Total carbon (TC)	[mg/gTS]	590 ± 14	638 ± 19	334 ± 14
Total organic carbon (TOC)	[mg/gTS]	575 ± 18	582 ± 17	310 ± 11
Total nitrogen (TN)	[mg/gTS]	92.2 ± 5.8	98.1 ± 5.1	35.7 ± 3.6
Total phosphorus (TP)	[mg/gTS]	4.0 ± 1.2	6.6 ± 1.2	1.9 ± 0.1
C/N ratio	-	6.4 ± 0.1	6.5 ± 0.1	9.35 ± 0.2
Protein	[%TS]	57.6 ± 3.6	61.3 ± 3.2	22.3 ± 2.2
Lipids	[%TS]	13.6 ± 3.1	12.7 ± 1.9	4.1 ± 0.7
Carbohydrates	[%TS]	16.1 ± 2.2	18.5 ± 1.3	2.3 ± 0.5

## Data Availability

Not applicable.

## Abbreviations

AD	anaerobic digestion
AGS	aerobic granular sludge
AS	anaerobic sludge
CAS	conventional activated sludge
CFC	closed fermentation chamber
CH_4_	methane
COD	chemical oxygen demand
CO_2_	carbon dioxide
DF	dark fermentation
EU	European Union
HRT	hydraulic retention time
H_2_	biohydrogen
MS	mineral dry matter
OLR	organic load rate
PF	photofermetation
SBR	sequencing batch reactor
SCO_2_	solidified carbon dioxide
TC	total carbon
TN	total nitrogen
TOC	total organic carbon
TP	total phosphorus
TS	dry matter
USA	United States of America
VS	organic dry matter
